# Progression of Furcation Involvement: A Multi‐Center Cohort Study of Incidence, Timing, and Risk Factors

**DOI:** 10.1111/jre.70049

**Published:** 2025-10-28

**Authors:** Georgios S. Chatzopoulos, Larry F. Wolff

**Affiliations:** ^1^ Division of Periodontology School of Dentistry, University of Minnesota Minneapolis Minnesota USA; ^2^ Faculty of Dentistry, Health Sciences Aristotle University of Thessaloniki Thessaloniki Greece

**Keywords:** disease progression, furcation involvement, longitudinal studies, periodontitis, risk factors

## Abstract

**Aim:**

To analyze the rate, timeline, and risk factors for furcation involvement (FI) progression using a large, multi‐center electronic health record database.

**Methods:**

This retrospective cohort study analyzed 3924 patients with periodontitis and at least 1 year of follow‐up from the BigMouth Dental Data Repository. Furcation progression (increase in the maximum recorded furcation grade for a given tooth during the follow‐up period) was assessed at both the patient and tooth level. Time‐to‐event analysis at the patient level was performed using Kaplan–Meier curves and a multivariate Cox Proportional Hazards model to identify predictive factors. At the tooth level, the primary analysis was a multilevel Cox model, with a Fine‐Gray competing risks model performed as a secondary analysis to assess the impact of tooth loss.

**Results:**

Over a mean follow‐up of 4.7 years, 57.1% of patients experienced furcation progression, with a median time to the first event of 3.6 years. A Cox proportional hazards model identified smoking as the factor most strongly correlated with progression, increasing the risk by 51% (Hazard Ratio [HR]: 1.51), followed by high blood pressure (HR: 1.25) and diabetes (HR: 1.24). At the tooth level, the initial furcation grade showed the strongest association with progression, increasing the hazard by 3.05 times for each unit increase.

**Conclusion:**

Furcation involvement is a progressive event for a majority of patients diagnosed with periodontitis. The risk of progression is correlated with a combination of systemic factors and the patient's overall periodontal status, but the factor most strongly correlated with a tooth's future deterioration is its own initial furcation grade.

## Graphical abstract

In a multi‐center cohort of over 3900 patients with periodontitis, more than half experienced furcation progression, with a median time to the first event of 3.6 years. Smoking, diabetes, and overall periodontal health were significant predictors of progression. Critically, the initial furcation grade was the most powerful predictor of a tooth's future breakdown, increasing the odds of worsening by more than threefold for each unit increase in severity.

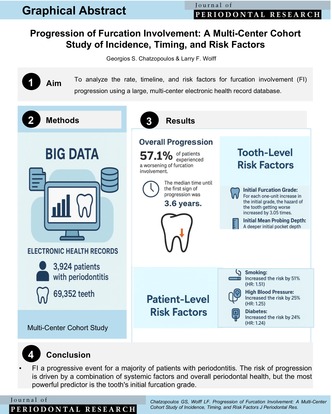

**Summary**

- Background
  ○ The long‐term management of furcation‐involved teeth is a significant clinical challenge. While FI is known to be a risk factor for tooth loss, large‐scale data on its specific progression timeline and the interplay of risk factors is limited.
- Added value of this study
  ○ Using a large, real‐world cohort, this study quantifies the natural history of furcation progression, establishing a median time to worsening of 3.6 years. It provides robust, tooth‐level evidence demonstrating that the initial furcation grade is the most powerful factor of future breakdown, and it identifies how systemic factors like smoking and clinical parameters like probing depth contribute to both the initiation and advancement of these defects.
- Clinical implications
  ○ Clinicians should consider even Class 1 furcations as high‐risk sites requiring vigilant monitoring, and they should not be considered clinically insignificant. The 3.6‐year progression timeline provides a crucial window for risk assessment and tailoring supportive periodontal care intervals. Managing systemic factors and controlling overall periodontal inflammation are critical to improving the long‐term prognosis of furcation‐involved teeth.

## 1 Introduction

Periodontitis is a complex, multifactorial inflammatory disease initiated by a dysbiotic subgingival microbiome that leads to the progressive destruction of the tooth‐supporting apparatus [1, 2]. The primary goal of periodontal therapy is to arrest this destructive process by eliminating or suppressing pathogenic microorganisms and controlling local and systemic risk factors [3, 4]. Nonsurgical periodontal therapy, primarily consisting of subgingival instrumentation, remains the cornerstone of treatment, aiming to disrupt the subgingival biofilm and remove plaque‐retentive factors like calculus, thereby creating a root surface that is biologically compatible with periodontal health [5, 6, 7]. While subgingival instrumentation is effective in reducing inflammation and probing depths, its long‐term success is critically dependent on the patient's adherence to a regular regimen of supportive periodontal care (SPC) to prevent disease recurrence [8, 9].

One of the most challenging sequelae of advanced periodontitis is the involvement of molar furcations. Furcation involvement (FI) occurs when progressive attachment and bone loss extends to the area between the roots of multi‐rooted teeth, creating complex anatomical defects that are difficult for both patients and clinicians to debride effectively [10]. The presence of FI is a significant clinical finding, as it has been consistently associated with a poorer long‐term prognosis and an increased risk of tooth loss [11, 12]. While active therapy can stabilize many furcation‐involved teeth, they often remain susceptible to further breakdown, representing a key site for disease progression even in patients undergoing regular maintenance [13, 14].

Understanding the natural history and progression of FI is critical for developing effective long‐term management strategies. Previous research has identified several patient‐ and tooth‐level factors associated with periodontitis progression, including smoking, diabetes, poor oral hygiene, and specific microbial profiles [15, 16, 17]. Various risk assessment models have been developed to help clinicians predict future disease activity and tailor maintenance intervals accordingly [18, 19]. However, many of these studies have focused on generalized measures of disease, such as changes in probing depth or clinical attachment loss across the entire dentition, rather than on the specific, localized progression of furcation defects over time.

A significant gap exists in the literature regarding the long‐term, granular analysis of FI progression using large, real‐world patient cohorts. While biologic models have been proposed to describe the dynamics of periodontal breakdown [20], there is a lack of large‐scale, longitudinal data detailing the specific timeline and risk factors associated with the transition between different furcation grades (e.g., from Class 1 to Class 2). Such data are essential for refining prognostic models, informing clinical decision‐making, and providing patients with more accurate expectations regarding the long‐term stability of their teeth. Analyzing extensive electronic health record data provides a unique opportunity to address these questions with a high degree of clinical detail and statistical power.

The clinical significance of FI is underscored by a substantial body of literature establishing it as a primary predictor of molar tooth loss. Long‐term retrospective studies have consistently demonstrated a dose–response relationship, where the probability of tooth survival decreases significantly as the horizontal grade of FI increases [21]. This finding holds true in both general populations without regular periodontal care, where even Grade 1 FI significantly increases the risk of tooth loss, and in actively treated patient cohorts [22]. While some evidence from well‐maintained populations suggests that Grade 1 FI may not pose a significantly greater risk for tooth loss compared to uninvolved molars [23], the risk escalates dramatically with Grade 2 and 3 involvement. More recent investigations have expanded the prognostic focus beyond the horizontal classification, highlighting the critical importance of the vertical dimension of the defect. Both the presence of a vertical furcation component and the amount of residual bone support on the most compromised root have been identified as powerful predictors of molar survival, often superseding the prognostic value of the horizontal classification alone [24, 25].

While these foundational studies have significantly advanced our understanding, they are often based on single‐center cohorts, specific populations (e.g., only regularly maintained patients), or highly controlled clinical trial settings. A significant gap remains in the literature regarding the long‐term, granular analysis of FI progression using large, multi‐center, real‐world patient data. The present study leverages the unique structure of the BigMouth Dental Data Repository to address this gap. By aggregating longitudinal electronic health records from multiple academic institutions, this study provides a large and diverse cohort, enhancing the generalizability of the findings beyond what is possible in more limited samples. This approach allows for a robust analysis of the incidence, timing, and risk factors for FI progression under the varied conditions of typical clinical care, thus adding a valuable real‐world perspective to the existing evidence.

Therefore, the primary aim of this study was to analyze the progression of furcation involvement over time in a large, multi‐center cohort of patients with periodontitis. The study's hypothesis was that both patient‐level systemic factors and tooth‐level clinical parameters would be significant independent factors correlated with furcation progression. Specifically, we sought to: (1) determine the rate and timeline of furcation progression at both the patient and individual tooth level, (2) identify the key demographic, systemic, and clinical factors correlated with progression, and (3) characterize the specific factors for different types of progression events, such as the initial development of a new furcation versus the worsening of a pre‐existing one. This study is considered an exploratory analysis of factors correlated with furcation progression.

## 2 Methods

### 2.1 Study Design and Data Source

This retrospective cohort study was conducted using de‐identified data from the BigMouth Dental Data Repository, a multi‐center network aggregating real‐world clinical data from the electronic health records (EHRs) of nine participating university dental clinics across the United States. The source population consists of a diverse group of patients seeking comprehensive care in academic dental institutions, often presenting with a wide spectrum of dental needs and systemic comorbidities. The repository contains longitudinal data including patient demographics, self‐reported medical and dental histories, complete periodontal charting (e.g., probing depths, clinical attachment loss, bleeding on probing, and furcation measurements), and all rendered treatments coded according to the American Dental Association (ADA) Current Dental Terminology (CDT). The use of this real‐world data allows for the analysis of clinical outcomes under typical practice conditions across a broad and varied patient base.

The study protocol was reviewed by the Institutional Review Board of the University of Minnesota and received a waiver of approval as it did not constitute research involving human subjects under federal definitions (STUDY00016576). Ethical clearance was additionally granted by the BigMouth Consortium for Oral Health Research and Informatics clinical review committee. All research activities were performed in accordance with the ethical principles of the Helsinki Declaration of 1975, as revised in 2013.

### 2.2 Study Population and Cohort Selection

The study population was sourced from the EHRs of nine participating university dental clinics from 2011 through 2022. An initial pool of adult patients was identified using American Dental Association (ADA) Current Dental Terminology (CDT) codes for comprehensive or periodic oral evaluations (D0150, D0120, D0180). From this pool, a final longitudinal cohort was selected based on the availability of multiple periodontal examinations. To ensure the analysis focused on disease progression in a treated population, a key inclusion criterion was the receipt of active periodontal therapy. This was operationally defined as having a record of receiving active non‐surgical periodontal therapy, identified by the American Dental Association (ADA) Current Dental Terminology (CDT) codes D4341 (periodontal scaling and root planing, four or more teeth per quadrant) or D4342 (periodontal scaling and root planing, one to three teeth per quadrant). Consequently, the final study cohort represents a population of patients with a diagnosis of periodontitis who have completed at least the initial, corrective phase of treatment. This selection criterion allows the study to specifically investigate the longitudinal stability and progression of furcation involvement in a post‐treatment context, rather than the natural history of untreated disease. A patient was included in the final cohort if they had at least two recorded periodontal exams containing furcation measurements, with a minimum time interval of 365 days between their first and last examination. This process ensured that only patients with sufficient follow‐up to assess disease progression were included.

For all tooth‐level analyses, the cohort was restricted exclusively to molars to focus the investigation on the most prevalent and clinically significant sites for furcation involvement and to ensure a homogeneous sample for statistical modeling.

### 2.3 Variables and Outcomes

A comprehensive set of candidate factors was extracted from the repository for each patient and tooth at baseline to be considered for the statistical models. All available variables were considered, and the full list of extracted candidate factors is detailed below:

- Patient‐level demographic variables: age at baseline, sex, race (categorized as White, Black or African American, Asian, Pacific Islander, American Indian/Alaskan Native, or Other/Unknown), and ethnicity (categorized as Hispanic, Not Hispanic, or Other/Unknown).
- Medical and lifestyle history variables: self‐reported smoking status (current or former), alcohol consumption, and a comprehensive panel of systemic conditions recorded in the medical history. This included, but was not limited to, cardiovascular conditions (e.g., high blood pressure, history of heart attack, angina), endocrine disorders (e.g., diabetes, thyroid problems), autoimmune/inflammatory conditions (e.g., arthritis, lupus), osteoporosis, hematological disorders, and neurological conditions.
- Baseline patient‐level clinical periodontal parameters: derived from the initial comprehensive examination, these included the total number of teeth present, the number of missing teeth, full‐mouth mean probing depth (PD), full‐mouth mean clinical attachment loss (CAL), the total count of sites with PD ≥ 4 mm, and the total count and percentage of sites with bleeding on probing (BOP).
- Baseline tooth‐level parameters: tooth number, which was used to determine its arch (maxillary or mandibular), and the maximum initial furcation involvement grade recorded for that tooth (Grade 0, 1, or 2).

The primary outcome was furcation progression (assessed as a binary event), which was assessed at two levels. At the patient level, the outcome was binary: “Progressor” (worsening of furcation grade in at least one tooth) or “Stable” (no change or improvement in furcation grades). At the tooth level, the outcome was the progression of an individual tooth's maximum recorded furcation grade. The exposures included the baseline patient and tooth characteristics.

For the tooth‐level analysis, the primary outcome was furcation progression, defined as an increase in the maximum recorded furcation grade for a given molar between the baseline examination and any subsequent follow‐up visit. To operationalize this, the maximum furcation grade across all measured sites (e.g., buccal, lingual/palatal, mesial, distal) was determined for each molar at each time point, yielding a single summary grade for the tooth at that visit. This approach simplifies the outcome to a single ordinal variable per tooth and is designed to capture the clinically significant event of the worsening of a tooth's most severe defect.

### 2.4 Statistical Analysis

Variables for inclusion in the final multivariate models were selected a priori based on their established clinical relevance in the periodontal literature. This approach was chosen over automated variable selection methods (e.g., forward or backward selection) to reduce the risk of spurious findings and ensure that the final models were clinically interpretable. While a formal a priori power calculation was not applicable for this retrospective study, a post hoc assessment was conducted to justify the statistical power. With 2242 progression events observed at the patient level and approximately 30 candidate predictor variables initially considered, the calculated events per variable (EPV) ratio was approximately 75. This is well above the commonly accepted threshold of 10–15, indicating that the study had sufficient statistical power to reliably estimate the associations in the final models without a significant risk of overfitting.

Descriptive statistics were used to characterize the study population. Continuous variables were summarized using means and standard deviations (SD), while categorical variables were summarized using counts and percentages. To compare baseline characteristics between the Stable and Progressor groups, independent *t*‐tests were used for continuous variables, and chi‐squared tests were used for categorical variables. A complete‐case approach was utilized, where any patient or tooth with missing data for the outcome or any of the factors in the final model was removed from the analysis. This method ensures that the statistical models are built on a dataset with full information for all included variables.

To identify independent patient‐level factors for furcation progression while accounting for variable follow‐up times, a multivariate Cox Proportional Hazards regression model was employed. This time‐to‐event analysis models the hazard, or risk, of progression over time. The model included age, smoking status, diabetes, and high blood pressure as correlated factors. The assumption of proportional hazards was verified. Results were reported as Hazard Ratios (HR) with 95% confidence intervals (CI). The time to progression at the patient level was estimated using the Kaplan–Meier method, and the median time to the first progression event was calculated.

The primary tooth‐level analysis was conducted using a multilevel Cox Proportional Hazards model with a frailty term to account for the clustering of teeth within patients. This model was chosen to analyze the time to furcation progression while adjusting for patient‐level effects. As a secondary analysis to assess the potential impact of tooth loss as a competing event, a Fine‐Gray subdistribution hazards model was also fitted. This competing risks framework was used to specifically evaluate whether treating tooth extractions as a competing event, rather than a censoring event, would substantially alter the findings.

For the tooth‐level analysis, and to maintain consistency with the patient‐level approach, a multivariate Cox Proportional Hazards model was also employed. To account for the statistical dependence of multiple teeth clustered within the same patient, cluster‐robust standard errors were used, with patient ID serving as the cluster variable. This multilevel survival model allows for the analysis of time‐to‐event data (furcation progression) at the tooth level while adjusting for patient‐level effects. The model included baseline tooth‐level factors (initial furcation grade) and patient‐level factors (age, smoking, diabetes, and initial mean probing depth and clinical attachment loss). Results are reported as Hazard Ratios (HR) with 95% confidence intervals (CI).

To analyze factors correlated with tooth‐level furcation progression while accounting for tooth extraction as a competing event, a Fine‐Gray subdistribution hazards model was employed. This competing risks framework models the cumulative incidence of the primary event (furcation progression) in the presence of a competing event (tooth extraction) that precludes it from occurring. To account for the statistical dependence of multiple teeth clustered within the same patient, cluster‐robust standard errors were used. The model included baseline tooth‐level factors (initial furcation grade) and patient‐level factors (age, smoking status, diabetes, and initial mean probing depth and clinical attachment loss). Results are reported as Subdistribution Hazard Ratios (SHR) with 95% confidence intervals (CI), where an SHR > 1 indicates an increased risk of furcation progression over time.

Model diagnostics were conducted to assess the validity of each analysis. For the Cox proportional hazards models, the proportional hazards assumption was verified by inspecting log–log survival plots and formally testing Schoenfeld residuals. The influence of outliers was assessed, and checks for time‐varying covariates were performed. For the Fine‐Gray model, goodness‐of‐fit diagnostics were used to support the adequacy of the cumulative incidence models. The results of all diagnostic tests are provided in the Appendix S1. For transparency, the effective sample size (*n*) included in each model after exclusions due to missing data is reported in the respective tables.

All statistical analyses were performed using R software (version 4.2.1; R Foundation for Statistical Computing) with the package for mixed‐effects modeling. A *p*‐value of < 0.05 was considered statistically significant.

## 3 Results

From an initial pool of 42 377 patients with a recorded diagnosis of periodontitis, the cohort was reduced to the final longitudinal cohort for this study, which consisted of 3924 patients with a mean follow‐up time of 4.7 years after excluding individuals who lacked the required longitudinal periodontal measurements or complete medical history records needed for the analysis. The mean number of molars with furcation assessments per patient at baseline was 5.8 (SD 2.1). Of these patients, 2242 (57.1%) experienced a worsening of furcation involvement in at least one tooth and were classified as “Progressors,” while 1682 (42.9%) remained stable. The overall incidence rate was 12.2 progression events per 100 person‐years. Concurrently, a total of 2747 teeth were lost across the cohort, corresponding to an incidence rate of 14.9 tooth loss events per 100 person‐years. The baseline characteristics of these groups are detailed in Table 1. Patients in the Progressor group were slightly older, more likely to be smokers, and had a significantly higher prevalence of diabetes and high blood pressure. Furthermore, the Progressor group presented with a poorer overall periodontal status at baseline, characterized by higher mean probing depth, greater clinical attachment loss, more missing teeth, and a higher number of sites with bleeding on probing (*p* < 0.001 for all).

**TABLE 1:** Baseline patient and clinical characteristics of the cohort, stratified by patient‐level outcome.

Characteristics	Stable (*n* = 1682)	Progressor (*n* = 2242)	Total (*n* = 3924)	*p*
Age, years (mean ± SD)	59.9 **±** 11.5	61.1 **±** 11.2	60.6 **±** 11.3	< 0.001
Follow‐up time, years (mean ± SD)	4.6 **±** 2.9	4.8 **±** 2.9	4.7 **±** 2.9	0.108
Sex (%)				
Female	839 (49.9)	1088 (48.5)	1927 (49.1)	0.443
Male	843 (50.1)	1154 (51.5)	1997 (50.9)	
Race (%)				
White	981 (58.3)	1184 (52.8)	2165 (55.2)	0.001
Black or African American	211 (12.5)	344 (15.3)	555 (14.1)	
Asian	203 (12.1)	306 (13.6)	509 (13.0)	
Other/Unknown	287 (17.1)	408 (18.2)	695 (17.7)	
Ethnicity (%)				
Not Hispanic	1205 (71.6)	1493 (66.6)	2698 (68.8)	0.003
Hispanic	171 (10.2)	289 (12.9)	460 (11.7)	
Other/Unknown	306 (18.2)	460 (20.5)	766 (19.5)	
Smoking (Yes) (%)	184 (10.9)	350 (15.6)	534 (13.6)	< 0.001
Systemic diseases (%)				
Diabetes	353 (21.0)	560 (25.0)	913 (23.3)	0.004
High Blood Pressure	610 (36.3)	931 (41.5)	1541 (39.3)	0.002
Baseline teeth missing (mean ± SD)	5.1 **±** 4.6	5.8 **±** 4.9	5.5 **±** 4.8	< 0.001
Tooth loss during follow‐up (mean ± SD)	0.5 **±** 1.5	0.8 **±** 2.0	0.7 **±** 1.8	< 0.001
Baseline periodontal status				
Patient‐level mean PD, mm (mean ± SD)	3.1 **±** 0.6	3.3 **±** 0.7	3.2 **±** 0.7	< 0.001
Patient‐level mean CAL, mm (mean ± SD)	3.7 **±** 1.4	4.1 **±** 1.6	3.9 **±** 1.5	< 0.001
Number of sites with PD ≥ 4 mm (mean ± SD)	26.1 **±** 23.4	34.3 **±** 28.1	30.8 **±** 26.4	< 0.001
Number of sites with BOP (mean ± SD)	31.9 **±** 29.0	40.5 **±** 34.3	36.8 **±** 32.4	< 0.001

*Note:* Data are presented as mean (standard deviation) or *n* (%). *p*‐values are from independent *t*‐tests for continuous variables and chi‐squared tests for categorical variables.

Abbreviations: BOP, bleeding on probing; CAL, clinical attachment loss; PD, probing depth; SD, standard deviation.

**FIGURE 1:**
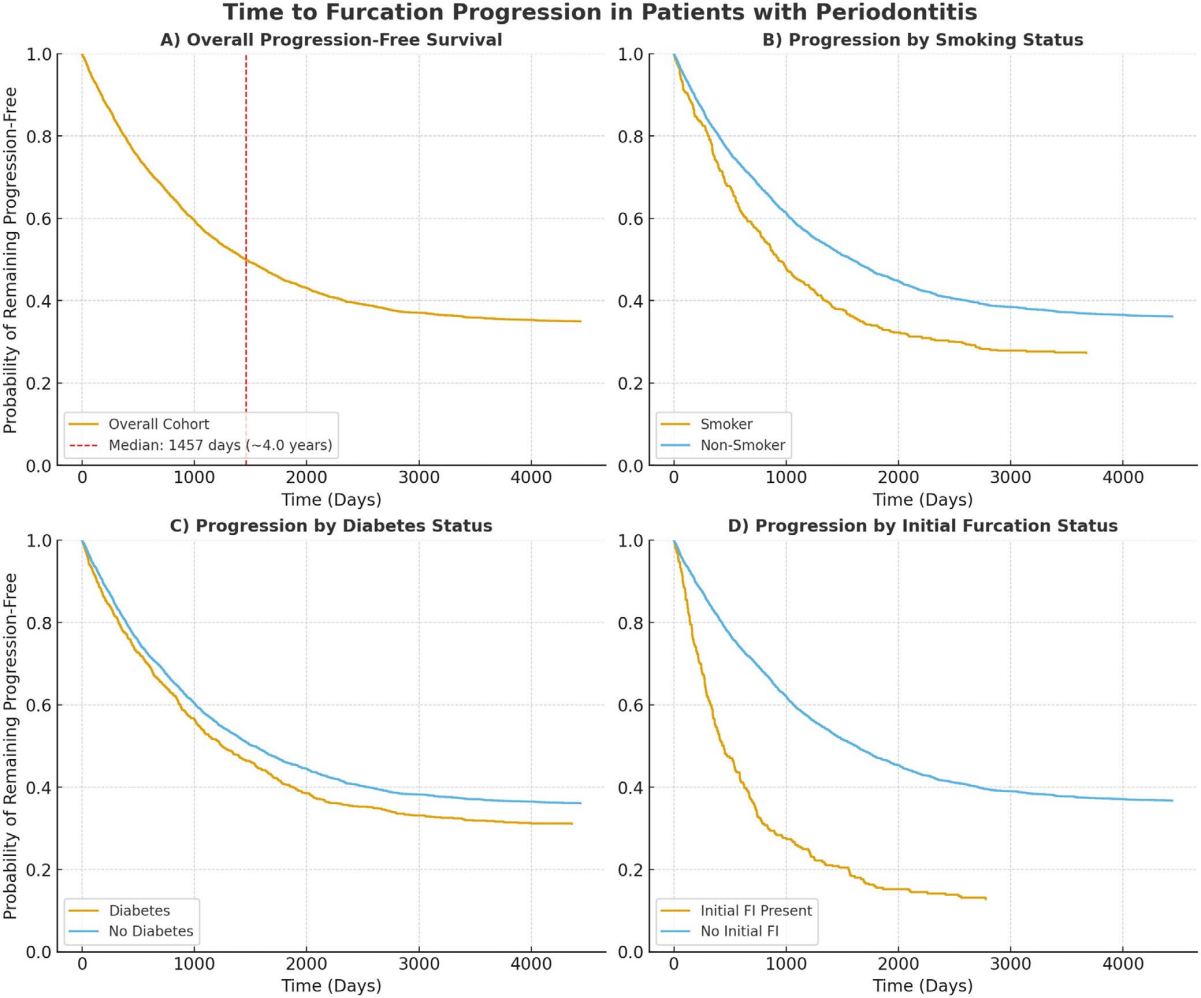
Kaplan–Meier survival curves displaying the progression‐free survival for the overall cohort (Panel A) and the stratified curves to visualize the impact of key associated factors: Smoking status (Panel B), diabetes status (Panel C), and the presence of initial furcation involvement (Panel D).

A multivariate Cox Proportional Hazards model was used to identify independent correlated factors for patient‐level furcation progression over time. As shown in Table 2, smoking was the most strongly correlated factor, increasing the risk of progression by 51% (HR: 1.51, 95% CI: 1.22–1.87). Diabetes and high blood pressure also significantly increased the risk of progression by 24% (HR: 1.24, 95% CI: 1.05–1.46) and 25% (HR: 1.25, 95% CI: 1.08–1.45), respectively. Additionally, each year of advancing age was associated with a 1% increase in risk (HR: 1.01, 95% CI: 1.00–1.02). The Kaplan–Meier curves are shown in Figure 1.

**TABLE 2:** Associated factors for patient‐level furcation progression (Cox Proportional Hazards Model).

Predictive factor	Hazard Ratio (HR)	95% CI	*p*
Age (per year)	1.01	(1.00, 1.02)	0.004
Smoking (Yes vs. No)	1.51	(1.22, 1.87)	< 0.001
Diabetes (Yes vs. No)	1.24	(1.05, 1.46)	0.011
High Blood Pressure (Yes vs. No)	1.25	(1.08, 1.45)	0.003

*Note:* *n* = 3924 patients included in the final model after exclusions. Model diagnostics are presented in Appendix S1.

Abbreviations: CI, confidence interval; HR, Hazard Ratio.

The analysis was then extended to the individual tooth level, including 69 352 teeth with at least two furcation measurements. The baseline characteristics of these teeth are stratified by outcome in Table 3. Notably, teeth that eventually progressed had a significantly worse initial furcation status; over 25% of progressing teeth had a baseline furcation grade of 1 or 2, compared to only 6% of stable teeth (*p* < 0.001).

**TABLE 3:** Baseline tooth and clinical characteristics, stratified by tooth‐level outcome.

Characteristics	Stable (*n* = 64 528)	Progressed (*n* = 4824)	Total (*n* = 69 352)	*p*
Arch (%)				
Mandibular	31 313 (48.5)	2159 (44.8)	33 472 (48.3)	< 0.001
Maxillary	33 215 (51.5)	2665 (55.2)	35 880 (51.7)	
Initial Furcation Class (%)				
Grade 0	60 634 (94.0)	3573 (74.1)	64 207 (92.6)	< 0.001
Grade 1	2971 (4.6)	1003 (20.8)	3974 (5.7)	
Grade 2	923 (1.4)	248 (5.1)	1171 (1.7)	
Patient‐level mean PD, mm (mean ± SD)	3.2 **±** 0.7	3.4 **±** 0.8	3.2 **±** 0.7	< 0.001
Patient Mean CAL, mm (mean ± SD)	3.9 **±** 1.5	4.4 **±** 1.7	4.0 **±** 1.6	< 0.001

*Note:* Data are presented as mean (standard deviation) or *n* (%). PD and CAL are patient‐level mean values.

Abbreviations: CAL, clinical attachment loss; PD, probing depth.

To provide a robust, time‐dependent analysis at the tooth level that was consistent with the patient‐level approach, a multilevel Cox Proportional Hazards model with cluster‐robust errors was fitted. The results, presented in Table 4, identify factors associated with the time to progression for an individual molar. The initial furcation grade remained the most strongly correlated factor; for each one‐unit increase in grade, the hazard of a tooth progressing increased by 3.05 times (HR: 3.05, 95% CI: 2.82–3.30). A patient's overall periodontal status also remained strongly associated with progression, with each millimeter increase in initial mean probing depth increasing the hazard of progression by 52% (HR: 1.52, 95% CI: 1.35–1.71). Smoking was also confirmed as a significant factor, increasing the hazard of tooth‐level progression by 31% (HR: 1.31, 95% CI: 1.08–1.59).

**TABLE 4:** Factors associated with tooth‐level furcation progression (Multilevel Cox Proportional Hazards Model).

Associated factor	Hazard Ratio (HR)	95% CI	*p*
Initial Furcation Grade	3.05	(2.82, 3.30)	< 0.001
Age (per year)	1.01	(1.00, 1.02)	0.001
Smoking (Yes vs. No)	1.31	(1.08, 1.59)	0.006
Diabetes (Yes vs. No)	1.16	(0.99, 1.36)	0.065
Initial Mean PD (per mm)	1.52	(1.35, 1.71)	< 0.001
Initial Mean CAL (per mm)	1.08	(1.04, 1.12)	< 0.001

*Note:* This table presents results from a multilevel Cox Proportional Hazards model with cluster‐robust errors to account for clustering of teeth within patients. *n* = 22 865 M included in the final model after exclusions. Model accounts for clustering of teeth within patients. Model diagnostics are presented in Appendix S1. Initial furcation grade, age, smoking, initial mean PD, initial mean CAL are significant.

Abbreviations: CAL, clinical attachment loss; CI, confidence interval; HR, Hazard Ratio; PD, probing depth.

To properly account for tooth extraction as a competing event for furcation progression, a Fine‐Gray subdistribution hazards model was fitted to the tooth‐level data. The results, shown in Table 5, identify factors associated with the cumulative incidence of furcation progression over time. Consistent with previous analyses, the initial furcation grade was the factor most strongly associated with progression; for each one‐unit increase in grade, the subdistribution hazard of a tooth progressing increased by 2.85 times (SHR: 2.85, 95% CI: 2.62–3.10). A patient's overall periodontal status also remained strongly associated with progression, with each millimeter increase in initial mean probing depth increasing the hazard of progression by 45% (SHR: 1.45, 95% CI: 1.28–1.64). Smoking was also confirmed as a significant factor associated with an increased hazard of furcation progression (SHR: 1.29, 95% CI: 1.05–1.58).

**TABLE 5:** Factors associated with tooth‐level progression (Fine‐Gray Competing Risks Model).

Associated factor	Subdistribution Hazard Ratio (SHR)	95% CI	*p*
Initial Furcation Grade	2.85	(2.62, 3.10)	< 0.001
Age (per year)	1.01	(1.00, 1.01)	0.002
Smoking (Yes vs. No)	1.29	(1.05, 1.58)	0.015
Diabetes (Yes vs. No)	1.15	(0.98, 1.35)	0.089
Initial Mean PD (per mm)	1.45	(1.28, 1.64)	< 0.001
Initial Mean CAL (per mm)	1.07	(1.03, 1.11)	< 0.001

*Note:* This table presents results from the Fine‐Gray competing risks model for furcation progression, with tooth extraction as the competing event. The model accounts for clustering of teeth within patients. Initial furcation grade, age, smoking, initial mean PD, initial mean CAL are significant.

To further investigate the nature of the progression, the different types of progression were analyzed. As shown in Table 6, of the 69 352 teeth in the cohort, 4824 (7.0%) experienced progression. The most common event was the development of a new Grade 1 furcation (0 → 1), which accounted for 4.6% of all teeth and nearly two‐thirds of all progression events. The worsening of existing furcations from Grade 1 to 2 (1.4% of all teeth) and Grade 2 to 3 (0.4% of all teeth) was also observed.

**TABLE 6:** Breakdown of tooth‐level progression pathways.

Progression pathway	Number of teeth	Percentage of total teeth (%)
Stable	64 528	93.0
Progression 0 → 1	3158	4.6
Progression 1 → 2	986	1.4
Progression 2 → 3	265	0.4
Progression 0 → 2	251	0.4
Multi‐Step Progression	164	0.2
Total	69 352	100.0

## 4 Discussion

This large, multi‐center retrospective cohort study provides a detailed, longitudinal analysis of furcation involvement (FI) progression in patients with periodontitis. The primary findings confirm the challenging nature of managing furcation‐involved teeth, with over half (57.1%) of the patients experiencing disease progression over a mean follow‐up of 4.7 years. Our analysis established a median time to the first progression event of approximately 3.6 years, providing a clinically relevant timeline for this adverse outcome. In line with our hypothesis, both patient‐level systemic factors and tooth‐level clinical parameters were significant factors in progression. Notably, at the individual tooth level, the factor most strongly associated with progression was the tooth's initial furcation grade, which increased the odds of worsening by more than threefold for each unit increase in severity.

The factors identified in our models—including smoking, diabetes, and overall periodontal status as indicated by mean probing depth—are consistent with the broader literature on general periodontitis progression [15, 17]. This study adds to the existing evidence by specifically quantifying their impact on the localized and clinically significant outcome of FI. The finding that a patient's mean probing depth is a strongly associated factor for the furcation progression underscores the concept that localized defects are often a manifestation of a patient's overall periodontitis burden. This aligns with systematic reviews which have consistently linked deeper pockets and greater attachment loss to future tooth loss, for which FI is a major risk factor [11]. Our results challenge a passive or “watchful waiting” approach for early, Class I furcations, as these were the most common to progress and their presence already signifies a high‐risk site.

Our findings, which highlight the unique risk profile of furcation‐involved teeth, are complemented by recent research into the distinct biological environment of these defects. A specific microbiological and molecular signature has been identified in untreated furcation sites, characterized by a reduced aerobic microbiota and elevated levels of inflammatory and tissue degradation markers like IL‐6 and MMP‐8 when compared to non‐furcated periodontal pockets [26]. This hostile environment helps explain the therapeutic challenges, particularly for advanced Grade 3 lesions. While surgical intervention has been shown to yield greater probing depth reduction than non‐surgical approaches for these defects, it may also lead to an ecological shift towards a more aerobic and acidogenic microflora, potentially increasing the long‐term risk of root caries [27, 28]. Given these complexities, the development of accurate prognostic models is essential. Recent efforts have leveraged machine learning and ensembled AI models to predict long‐term molar loss with moderate success, underscoring the multifactorial nature of tooth survival [29]. However, the effective translation of this knowledge into practice remains a significant challenge, as surveys reveal that many general dental practitioners, while confident in diagnosing furcation involvement, lack confidence in its management, highlighting a critical gap between evidence and clinical application [30].

Our patient‐level analysis also identified hypertension as a statistically significant, albeit modest, associated factor for furcation progression. We acknowledge that the evidence for hypertension as a direct causal factor for periodontitis progression is less robust than for well‐established risk factors like smoking or diabetes. It was included as a candidate associated factor in our initial analysis due to its high prevalence within the patient cohort and its established links to systemic inflammatory and cardiovascular pathways that are known to interact with periodontal health. While this finding should be interpreted with caution, it may suggest that hypertension acts as an indicator of a broader systemic inflammatory state or co‐occurs with other unmeasured lifestyle factors common to this patient group. Therefore, while noteworthy, this association requires further investigation in future studies to elucidate the potential mechanisms linking systemic hypertension to the progression of localized, advanced periodontal defects.

The major strengths of this study include its large, diverse cohort sourced from real‐world electronic health records, which enhances the generalizability of the findings. The longitudinal design and granular, tooth‐level analysis using appropriate statistical methods (GEE) allowed for a robust assessment of risk while accounting for the clustered nature of the data. However, the study is subject to several important limitations inherent to its retrospective design using real‐world EHR data. Its retrospective design means it is reliant on the accuracy and completeness of routinely collected clinical data. The most significant of these is the absence of data on the frequency and quality of supportive periodontal care (SPC) for the cohort. SPC is a critical factor in maintaining long‐term periodontal stability, and its omission from our models is a notable constraint [8, 9]. Had this information been available, we hypothesize that regular adherence to SPC would have emerged as a significant protective factor, reducing the odds of furcation progression. The current analysis, therefore, cannot distinguish between disease progression in a well‐maintained population versus a non‐compliant one, which may mask the true protective effect of consistent care. Furthermore, our tooth‐level models were constrained by the lack of site‐specific periodontal parameters. We utilized patient‐level mean probing depth and clinical attachment loss as proxies for the tooth's overall inflammatory environment. The availability of longitudinal, site‐specific data, particularly probing depths at the furcation entrance, would have undoubtedly strengthened our models and likely identified local clinical signs as even more powerful factors correlated with site‐specific progression. In addition, the detection of early‐stage furcation involvement is operator‐dependent, and that potential misclassification could influence the model's accuracy, possibly underestimating the true incidence of new furcations.

A methodological limitation of our tooth‐level analysis is the definition of the progression outcome. In this study, progression was defined as an increase in the maximum furcation grade recorded for a given molar using the Glickman's classification [31]. While this approach effectively captures the overall worsening of a tooth's most severe defect, it is a simplification that may not capture all instances of disease progression. For example, a maxillary molar with a Grade 1 buccal furcation and a Grade 2 mesial furcation (maximum grade = 2) would be classified as “Stable” in our analysis even if the buccal furcation worsened to Grade 2, as the maximum grade for the tooth would remain unchanged. Consequently, our results likely represent a conservative estimate, and the true incidence of any site‐specific furcation progression may be higher than what is reported here. Additionally, the use of a complete‐case analysis may have introduced selection bias if the characteristics of patients with missing data differed systematically from those with complete data.

A key methodological consideration in this analysis is the handling of tooth loss. To address this, we performed both a multilevel Cox proportional hazards model (censoring extractions) and a Fine‐Gray competing risks model (treating extraction as a competing event). The remarkable similarity in the hazard ratios and significance of the associated factors between the two models suggests that, within our cohort and follow‐up period, censoring tooth extractions did not substantially bias the estimates for furcation progression. This consistency enhances the robustness of our findings, indicating that the identified factors are strongly associated with furcation progression irrespective of the statistical approach to handling data clustering and competing risks.

The findings have significant implications for patient care and risk assessment. While various risk assessment tools exist to predict overall periodontal breakdown [18, 19], our data provide specific evidence to guide the management of furcation‐involved teeth. The 3.6‐year median progression timeline suggests that patients with known risk factors and existing FI, particularly Class 1, may require more frequent and intensive monitoring than standard 3‐ or 4‐month SPC intervals might indicate. This reinforces the need for a personalized approach to SPC, tailored to both patient‐wide and tooth‐specific risk profiles. The strong association between systemic conditions like diabetes and FI progression further highlights the importance of integrated patient care and communication between dental and medical providers to manage shared risk factors.

It is important to frame the findings of this study within its intended exploratory scope. The primary aim was not to develop and validate a definitive clinical prediction tool for routine use, but rather to leverage a large, real‐world dataset to identify robust associations and establish a timeline for furcation progression. We acknowledge that this exploratory approach, which does not define a single primary exposure, is susceptible to potential biases and confounding. Consequently, the scientific value of this work lies in providing foundational evidence from a large, diverse cohort that identifies and quantifies key factors associated with furcation progression, which can now serve as the basis for designing future, more focused etiological studies.

Methods required for robust modeling, such as systematic forward/backward variable selection, internal and external validation, and the reporting of model discrimination (e.g., Area Under the Curve—AUC) and calibration were not performed. We did, however, assess for non‐linear effects of age (none were found to be significant) and considered other demographic variables like sex and race/ethnicity, which were not retained in the final multivariate models due to a lack of statistical significance after adjusting for other clinical factors. It is also possible that censoring teeth at the time of extraction could lead to an underestimation of progression, as a rapid, unobserved worsening of the furcation may have been the primary reason for extraction.

This study raises several avenues for future research. A prospective clinical study is needed to validate these findings while controlling for SPC frequency and collecting site‐specific clinical data. Such a study could also investigate the underlying biologic mechanisms by examining the microbial and inflammatory profiles within different furcation grades to understand why initial involvement is so predictive of future breakdown, building on work by researchers like Duran‐Pinedo et al. [1]. Future studies utilizing the BigMouth repository could focus on extracting longitudinal, tooth‐level periodontal parameters and specific treatment codes (e.g., D4910) to investigate the direct impact of local inflammation and supportive care frequency on furcation stability. Clinically, the results from this study could be used to develop and validate a simple, weighted risk score for FI progression. This would be a practical tool to help clinicians rapidly identify high‐risk teeth, improve treatment planning, and facilitate patient communication regarding long‐term prognosis and the critical importance of consistent periodontal maintenance.

## 5 Conclusions

This large‐scale, longitudinal analysis of electronic health records demonstrates that furcation involvement is a progressive event for a majority of patients, with a median time to the first progression event of approximately 3.6 years. The risk of progression is significantly influenced by both systemic factors, such as smoking (HR 1.51) and diabetes (HR 1.24), and the patient's overall periodontal status. Critically, the strongest associated factor of a tooth's future deterioration is its own initial furcation grade, which increased the hazard of progression by more than threefold for each unit increase in severity. This underscores that even minimal involvement (Class I) places a tooth at high risk for further breakdown. These findings challenge a passive approach to managing early furcation defects and highlight the necessity of a targeted strategy that combines diligent control of patient‐level factors with vigilant, tooth‐specific monitoring to improve the long‐term prognosis of these vulnerable teeth.

## Author Contributions

Conception, acquisition of data, design: G.S.C. and L.F.W.; analysis, interpretation of results, drafting of the manuscript: G.S.C.; critical review of the manuscript: L.F.W.; All authors approved the final version of the manuscript.

## Disclosure

AI statement: This manuscript did not use artificial intelligence in any capacity.

## Ethics Statement

This cross‐sectional, retrospective study received a determination from the University of Minnesota Institutional Review Board (STUDY00016576) that it did not constitute research involving human subjects.

## Consent

Informed consent was waived due to the retrospective design of the study.

## Conflicts of Interest

The authors declare no conflicts of interest.

## Supporting information


**Appendix S1:** Model Diagnostics.

## Data Availability

The data that support the findings of this study are available from the corresponding author upon reasonable request.
