# Supplementary material for: Progression of Furcation Involvement: A Multi‐Center Cohort Study of Incidence, Timing, and Risk Factors
Source: J Periodontal Res. 2025 Oct 28;61(3):273–82. doi: 10.1111/jre.70049 (PMC13140782; doi:10.1111/jre.70049)
Supplement: Supplementary file 1 — Appendix S1: Model Diagnostics. [file JRE-61-273-s001.docx]

Appendix S1: Model Diagnostics

This appendix provides the results of the comprehensive diagnostic tests performed for the multivariate models presented in the main manuscript.

**1. Patient-Level Cox Proportional Hazards Model (Table 2)**

- **Proportional Hazards Assumption:** The proportional hazards assumption was assessed both visually and statistically. Visual inspection of log-log survival plots for each covariate (e.g., smoking, diabetes) showed approximately parallel lines between the groups, providing initial support for the assumption. The formal global test based on scaled Schoenfeld residuals was non-significant (p = 0.354), providing no statistical evidence to reject the proportional hazards assumption for the model.
- **Assessment of Influential Outliers:** The influence of individual observations on the model's coefficients was assessed by examining the deviance residuals. No highly influential outliers that would significantly alter the hazard ratios or overall conclusions were detected.
- **Time-Varying Covariates:** The model was tested for evidence of time-varying effects by creating and testing interaction terms between covariates and a function of time. None of these interaction terms were found to be statistically significant, suggesting the effect of the associated factors did not change over the follow-up period.
- **Model Fit:** The overall model demonstrated adequate fit for this exploratory analysis, with a significant likelihood ratio test (p < 0.001), indicating that the model including the associated factors was a significant improvement over a null model.

**2. Tooth-Level Multilevel Cox Proportional Hazards Model (Table 4)**

- **Proportional Hazards Assumption:** The proportional hazards assumption for the tooth-level model was also confirmed. The global test based on Schoenfeld residuals was non-significant (p = 0.218). Visual inspection of log-log survival curves stratified by key covariates (e.g., initial furcation grade, smoking status) showed parallel lines, supporting the assumption.
- **Assessment of Influential Outliers:** Examination of residuals did not identify any influential outliers or clusters of outliers that would disproportionately affect the model estimates.
- **Time-Varying Covariates:** As with the patient-level model, no evidence of significant time-varying effects was detected for the included covariates.

**3. Fine-Gray Competing Risks Model (Table 5)**

- **Goodness-of-Fit:** Goodness-of-fit diagnostics, including visual inspection of cumulative incidence function plots, were performed and supported the adequacy of the Fine-Gray model for this analysis.
- **Proportional Hazards Assumption:** The proportional subdistribution hazards assumption was visually inspected and deemed acceptable for this exploratory analysis, with no major deviations observed.

**4. Multicollinearity Assessment (All Models)**

- **Variance Inflation Factors (VIFs)** were calculated for the associated factors included in all models to assess for multicollinearity. All VIFs were well below the common threshold of 5 (all < 1.3), indicating that no significant multicollinearity was present among the variables in any of the fitted models.
